# Picoliter Cuvette inside an Optical Fiber to Track Gold Nanoparticle Aggregation for Measurement of Biomolecules

**DOI:** 10.3390/s19132859

**Published:** 2019-06-27

**Authors:** Masahiko Shiraishi, Kazuhiro Watanabe, Shoichi Kubodera

**Affiliations:** 1 Department of Information Systems Science, Faculty of Science and Engineering, Soka University, 1-236, Tangi-machi, Hachioji, Tokyo 192-8577, Japan; 2 Department of Science and Engineering for Sustainable Innovation, Faculty of Science and Engineering, Soka University, 1-236, Tangi-machi, Hachioji, Tokyo 192-8577, Japan

**Keywords:** optical fiber sensor, biological sensing, picoliter sensing capacity, femtosecond laser processing, gold nanoparticles, localized surface plasmon resonance

## Abstract

This study demonstrated a measurement approach for biomolecules at the picoliter scale, using a newly developed picoliter cuvette inside an optical fiber constructed via near-ultraviolet femtosecond laser drilling. The sensing capacity was estimated to be within 0.4–1.2 pL due to an optical path length of 3–5 microns, as measured by scanning electron microscopy (SEM). The picoliter cuvette exhibited a change in the optical extinction spectrum after addition of biomolecules such as L-cysteine, in conjunction with a gold nanoparticle (GNP) dispersion solution, following a simple measurement configuration involving a small white light source and a compact spectrometer. A linear attenuation of the spectral dip near a wavelength of 520 nm was observed as the L-cysteine concentration was increased at 4 wt% of the GNP mass concentration. The measurement resolution of the concentration using the picoliter cuvette was evaluated at 0.125 mM. The experimental results showed the difference in aggregation processes caused by a different concentration of GNPs. Moreover, they revealed the ability of the picoliter cuvette to verify whether the concentration of GNPs in the liquid sample correspondingly determines homogeneous or inhomogeneous GNP aggregation, as supported by SEM observation and numerical calculations based on Mie theory.

## 1. Introduction

There has been a remarkable advancement in technologies capable of recognizing cells or DNA indicators for the diagnosis of specific tumors and cancers [1,2,3,4]. For instance, the significant technological progress of the extraction of sensing targets—cells or DNA—has made the measurement of their trace amounts achievable [5,6]. Further, spectroscopic measurement of such targets affords a possibility for their discrimination and quantification at the end of the measurement process [7,8]. Moreover, with spectrometry, the sensing targets can be measured using an extremely small sample volume, which is an approach worth taking in research and practical medical sites considering the amount of valuable samples that could be saved. Thus, for one-time measurement of a liquid sample with a small amount of the sensing targets, both the absolute amount of sample and the target’s concentration must be necessarily low. An absolute small space is required for holding the extremely small liquid sample during the spectroscopic measurement. Given the difficulty of the liquid sample remaining as a droplet for a certain time in a picoliter scale, it is preferable for it to be surrounded by a wall, except for the inlet and outlet. In addition, the light utilized for the spectroscopic measurement must be irradiated to the liquid sample accurately, and accordingly, the light transmitted or scattered from the sample must be guided to the detectors efficiently. However, reducing the volume of the liquid sample for spectroscopic measurement simultaneously causes a weaker signal from the sensing targets. In this regard, a representative method of fluorescent staining that intentionally operates the signal for the sensing target’s capture was immediately conceived [9]. Recent methods of spectroscopic measurement have employed metal nanoparticles—gold nanoparticles (GNPs) in particular [10,11,12]. The GNPs were dispersed stably in a solution under room temperature and exhibited optical characteristics of a high-extinction coefficient in the visible region with nanometer particle size [13,14]. Additionally, GNPs were expected to show low toxicity with biomolecules and cells as their sensing targets [15], which, when added into the GNP dispersion solution to induce aggregation of the nanoparticles, results in a change in the optical extinction spectrum with the spectroscopic measurement [16,17,18,19,20]. On this basis, utilizing GNPs for spectroscopic measurement is effective, as measurement of the sensing targets becomes possible by tracking of the change in the optical extinction spectrum. Nonetheless, the abovementioned requirements are achievable with the provision of a small space inside a small diameter and lightweight optical fiber, and with the injection of the GNP dispersion solution into the space. Ablation processing by ultra-short pulse laser is effective in creating a space of a micrometer scale, at an arbitrary position, of a transparent material such as the optical fiber [21,22]. For instance, a micro through-hole was fabricated into a glass optical fiber without the material breaking off, by using femtosecond laser drilling [23]. Subsequently, optical extinction spectra peculiar to the GNPs were obtained by injection into the micro through-hole [24]. This suggests the feasibility of the micro through-hole as a picoliter cuvette inside an optical fiber, combined with GNP dispersion solution, as a technique for the measurement of biomolecules. This study realizes the biological sensing of L-cysteine by tracking the aggregation process of GNPs in a small liquid sample using a picoliter cuvette inside an optical fiber. The optical extinction spectra were obtained by injection of GNP dispersion solution, following a simple measurement configuration of a small white light source and a compact spectrometer. L-cysteine was added into the solution to induce GNP aggregation, and further, to induce the change in the optical extinction spectra based on localized surface plasmon resonance (LSPR). It was considered that the LSPR signal of extinction spectrum was red-shifted when GNPs with a large particle diameter contributed to the transmission signal due to aggregation. However, it was limited to the case where the particle diameter homogeneously increased and was abundant in a very short optical path length. When the initial size GNPs free from aggregation were abundant, the LSPR signal of the initial GNPs decreased due to a decrease in particle numbers due to aggregation. Biomolecular measurement was also possible by tracking the change in the optical extinction spectrum. Results of the scheme showed that the picoliter cuvette is capable of verifying whether GNPs in the liquid sample were homogeneously aggregated, or whether there was an abundant amount of GNPs avoiding such aggregation. These results were in good agreement with those of an observation that employed field emission scanning electron microscopy (FE-SEM) and numerical calculations based on Mie theory.

## 2. Materials and Methods

### 2.1. Fabrication of Picoliter Cuvette inside an Optical Fiber

**Figure 1:**
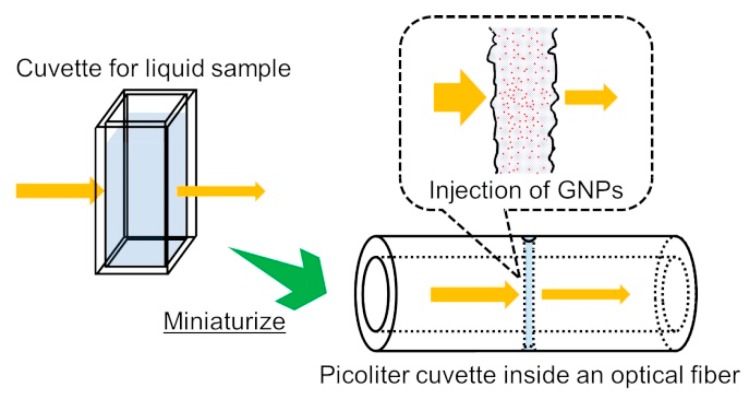
Conceptual diagram of a picoliter cuvette inside an optical fiber. Structure of the picoliter cuvette with a space penetrating the optical fiber.

Figure 1 shows a conceptual diagram of the picoliter cuvette inside a glass optical fiber, which was fabricated by femtosecond laser ablation processing. The volume of the retained liquid sample in the picoliter cuvette that was capable of interacting with the optical fiber propagating light was of a picoliter scale. The propagating light in the optical fiber was utilized for spectroscopic measurement following its efficient irradiation into the liquid sample, as well as the transmitted light being guided smoothly to a photo-detector after interaction with the liquid sample. The laser processing procedure to make the picoliter cuvette has been described in detail in a previous study [24]. Briefly, fabrication of the picoliter cuvette was performed at a wavelength of 400 nm, the second harmonic wavelength of a femtosecond laser system (IFRIT, Cyber Laser Inc., Tokyo, Japan), via a wavelength converter. 

Femtosecond pulses of 30 μJ pulse energy were focused on the multi-mode graded index glass optical fiber, with 62.5 and 125 μm core and cladding diameters, respectively, using a near-ultraviolet objective lens with a numerical aperture of 0.65. The pulse width and repetition rate of the femtosecond pulses were 350 fs and 1 kHz, respectively, and their focusing position was fixed at a 50 μm depth from the surface of the optical fiber. A total of 150 pulses were irradiated onto the optical fiber, succeeded by irradiation of the pulse train from the opposite side under the same conditions, with the formed micro through-hole. Based on the observation under FE-SEM (JSM-7500F, JEOL Ltd., Tokyo, Japan), the optical path length was evaluated at 3–5 μm. With the above conditions, the volume capable of interaction with the optical fiber propagating light and the liquid sample was estimated at 0.4–1.2 pL [24].

### 2.2. Acquisition of Optical Extinction Spectra

**Figure 2:**
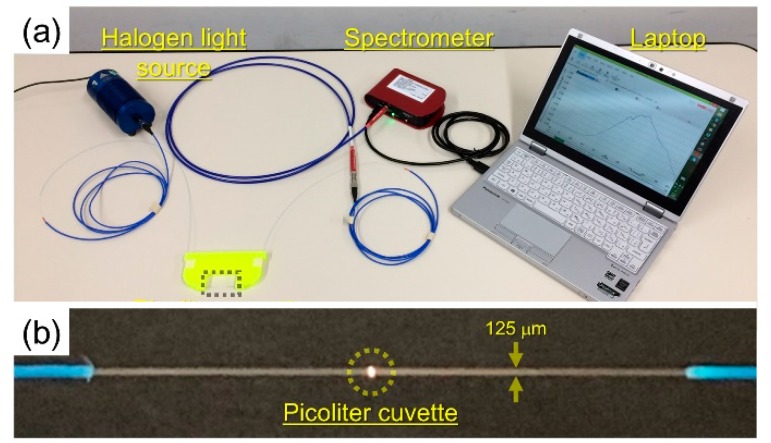
(**a**) Measurement configuration for acquisition of optical extinction spectra. The concentration of L-cysteine is measured with a simple configuration of a compact white light source, a compact spectrometer with a laptop for storing light intensity spectrum data, and the picoliter cuvette. (**b**) Enlarged picture of the picoliter cuvette inside the optical fiber, represented as a dotted square in (**a**). The optical fiber propagating light irradiated the cuvette, and the scattered light is placed in the photograph.

Figure 2a shows the measurement configuration in acquiring the optical extinction spectrum. For the measurement, a small halogen light source (HL-2000, Ocean Optics, Inc., Largo, FL, USA) and a compact spectrometer (CCS200/M, Thorlabs, Inc., Newton, NJ, USA) were used. Both ends of an optical fiber cable with the picoliter cuvette were connected to the light source and the spectrometer, respectively. Specifically, the spectrometer covered wavelengths of 400–800 nm, with 200 measurements averaged in the accumulative time of 3 ms each. Figure 2b shows an enlarged view of the picoliter cuvette. The optical fiber propagating light was irradiated into the picoliter cuvette, and the scattered light was displayed near the sensor portion of the photograph. GNP dispersion solution was purchased from Tanaka Kikinzoku Kogyo Co., Ltd. (Tokyo, Japan). GNPs were monodispersed in pure water with Polyvinylpyrrolidone (PVP) to prevent conjugation. Based on product specification, the GNPs had particle diameters of 5–10 nm, and an average particle diameter of 8.1 nm. The ratio of the light intensity spectrum obtained by injecting the GNP dispersion solution containing L-cysteine (Wako Pure Chemical Industries, Ltd., Osaka, Japan) was acquired with reference to the light intensity spectrum of pure water, as the output optical extinction spectrum of GNPs. In the experiment, L-cysteine was added to the GNP dispersion solution, and induced change in the optical extinction spectrum by the aggregation of GNPs was obtained. The thiol groups of L-cysteine adsorbed the surface of GNPs with high reactivity. Then, aggregation occurred due to hydrogen bonding by the combinations of the amino groups and/or carboxy groups of L-cysteine. The concentration of L-cysteine was set in the range of 0–5 mM, which was deemed sufficient to induce GNP aggregation by addition of L-cysteine. After the addition, the dispersion solution was allowed to stand for more than 3 h at room temperature. After the aggregation process stopped, the liquid sample was injected to fill the picoliter cuvette. The mass concentration of gold in the dispersion solution was set to 2 wt% and 4 wt%. The spectra were averaged with 10 measurements under the same concentrations of GNPs and L-cysteine.

### 2.3. Immobilization of GNPs for Scanning Electron Microscopy (SEM) Observation

GNPs were immobilized onto thin fused silica plates in order to observe the size of the particles. The surface of the plates was cleaned by ethanol and deionized (DI) water. After air blow drying, the plates had been immersed in a 1% 3-aminopropyltrimethoxy silane (3-APTMS) solution for 30 min to form a self-assembled monolayer (SAM) on the surfaces of plates. After that, the surface of the plates were dried in the heater for 2 h with a temperature of 110 °C. Then, the plates were immersed in GNP dispersion solution for 30 min, followed by rinsing with DI water and air blow drying. Since the aggregated GNPs used in the experiment were thick, the GNP dispersion solution was diluted by 1/100 in DI water.

## 3. Results and Discussion

**Figure 3:**
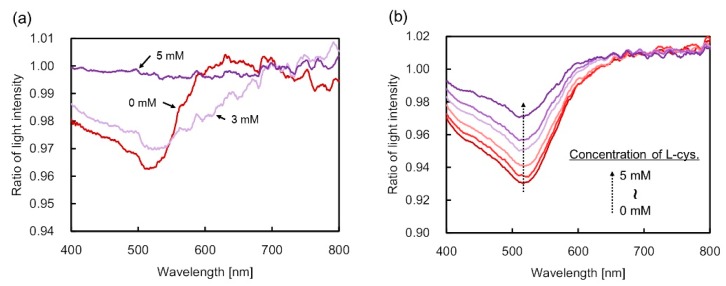
Optical extinction spectra acquired with the picoliter cuvette inside an optical fiber for changes in L-cysteine concentration. (**a**,**b**) are for gold mass concentration cases of 2 wt% and 4 wt%, respectively. The numerical value in the graph represents the concentration of L-cysteine.

**Figure 4:**
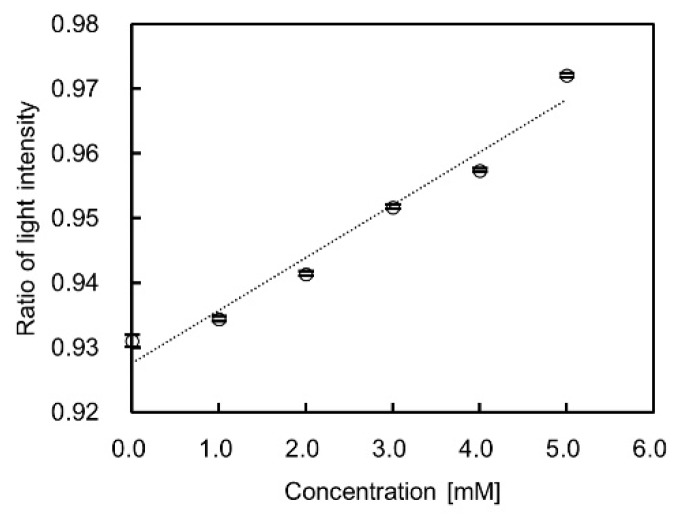
Sensitivity characteristics of L-cysteine using gold nanoparticle (GNP) dispersion solution with a gold mass concentration of 4 wt%. The white circle is the average value of ten measurements, and the bars show the maximum and minimum value in each plot.

Figure 3a shows the optical extinction spectra of the GNP dispersion solution fixed at 2 wt% with varied concentrations of L-cysteine. As the concentration was increased, the full-width at half-maximum of the bandwidth of the extinction spectrum broadened in the range of 0–5 mM. The dip of the spectrum decreased at more than 3 mM of L-cysteine concentration. Addition of L-cysteine caused a change in the optical extinction spectrum even when the mass concentration of GNPs was kept constant. More particularly, the change in the spectrum was attributed to the influence of GNP aggregation following the addition of L-cysteine. Moreover, the particle size of the GNPs in the dispersion solution became homogeneously larger than the initial size of 5–10 nm due to nanoparticle aggregation. Accordingly, the optical extinction spectra were obtained when the mass concentration of GNPs in the dispersion solution was 4 wt%, as shown in Figure 3b. Compared with the case of 2 wt%, the extinction dip in the vicinity of 520 nm decreased gradually. Wavelength shift did not occur due to the remaining large number of particle sizes in the initial size, which was subsequently reduced with the GNP aggregation. Similarly, the particle number of aggregated GNPs increased, but the particle number of the initial size remained high. Figure 4 illustrates the sensitivity characteristics with L-cysteine based on the 520 nm wavelength obtained from the experiments at 4 wt% of gold mass concentration. Based on the results, the change in the light intensity ratio as concentration increased was close to linear. Moreover, with the resolution of the light intensity output of the compact spectrometer for measurement assumed at 0.001, the measurement resolution of L-cysteine was 0.125 mM due to a sensitivity of 0.008 mM^−1^, as depicted in Figure 4.

**Figure 5:**
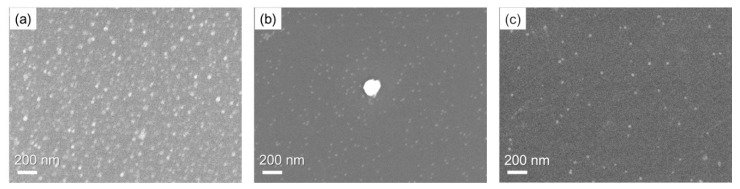
Scanning electron microscopy (SEM) photographs of GNPs after aggregation. (**a**) GNPs after addition of 2 mM L-cysteine into the dispersion solution of 2 wt% gold mass concentration. (**b**) GNPs after addition of 5 mM L-cysteine into the dispersion solution of 4 wt% gold mass concentration. (**c**) GNPs without L-cysteine into the dispersion solution of 4 wt% gold mass concentration.

Results for the observation of the GNPs were obtained with FE-SEM. Figure 5a shows a typical SEM photograph of the GNPs after aggregation at 2 wt% of gold mass concentration and 2 mM of L-cysteine concentration. As observed, the GNPs’ diameter was homogeneously larger than 10 nm, which is consistent with the expectation from the optical extinction spectra obtained in the experiment. Figure 5b depicts the GNPs at 4 wt% of gold mass concentration and 5 mM of L-cysteine concentration. The added L-cysteine caused aggregation of the GNPs, resulting in larger particle diameters with 200 nm as the major diameter, compared to the initial size of particles in the center of the photograph. Simultaneously, a number of particles were found to avoid aggregation—an observation which is consistent with the experimental result shown in Figure 3b. In order to confirm that the aggregation of GNP was due to the addition of L-cysteine, a SEM photograph of GNPs without L-cysteine is shown in Figure 5c. The aggregated GNPs with larger particles than the initial size were not identified.

Accordingly, Mie theory was applied for calculating the optical extinction spectrum to ascertain the reason for the variation in the spectrum brought about by the aggregation of nanoparticles after addition of L-cysteine. Since the size of the GNPs was on the order of tens of nanometers, any waves even inside the optical fiber could be approximated as a plane wave. This is one of the reasons why the calculation based on Mie theory reasonably reproduced the experimental results. The input size parameters for the analysis were based on particle absorption and scattering for the incident light [25]. The average size prior to aggregation was 8.1 nm. Nonetheless, due to the increase in particle diameter following aggregation, the absorption and scattering cross-sections based on the Rayleigh approximation were not applicable in the calculation. Thus, the optimum extinction spectrum was calculated using the extinction cross-section *C_ext_* from Mie theory, as a function of the wavelength *λ* [26]:

(1) Cext=λ22π∑n=1∞(2n+1)Re(an+bn).

The scattering coefficients *a_n_* and *b_n_* are as follows:

(2) $$a_{n}=\frac{m\psi_{n}\left(mx\right)\psi_{n}^{\prime }\left(x\right)-\psi_{n}\left(x\right)\psi_{n}^{\prime }\left(mx\right)}{m\psi_{n}\left(mx\right)\xi_{n}^{\prime }\left(x\right)-\xi_{n}\left(x\right)\psi_{n}^{\prime }\left(mx\right)},$$

(3) $$b_{n}=\frac{\psi_{n}\left(mx\right)\psi_{n}^{\prime }\left(x\right)-m\psi_{n}\left(x\right)\psi_{n}^{\prime }\left(mx\right)}{\psi_{n}\left(mx\right)\xi_{n}^{\prime }\left(x\right)-m\xi_{n}\left(x\right)\psi_{n}^{\prime }\left(mx\right)},$$

where *m* denotes the expression *m* = *n_p_*/*n_m_*; and *n_p_* and *n_m_* are the refractive indices of the particle and the medium, respectively. The refractive index of gold was calculated as a function of the wavelength using an approximate equation of the dielectric function [27]. Additionally, *x* in the scattering coefficients is a size parameter, expressed as *x* = 2*πn_m_a*/*λ*, where *a* is the radius of the nanoparticle. The parameters *ψ_n_*(*ρ*) and *ξ_n_*(*ρ*) are defined as follows:

(4) $$\psi_{n}\left(\rho \right)=\rho j_{n}\left(\rho \right),$$

(5) $$\xi_{n}\left(\rho \right)=\rho h_{n}^{\left(1\right)}\left(\rho \right),$$

*ψ_n_*(*ρ*) is the Riccati–Bessel function based on the first-order spherical vessel function *j_n_*(*ρ*), and similarly, *ξ_n_*(*ρ*) is the Riccati–Bessel function based on the first-class Hankel function *h_n_*^(1)^(*ρ*). Although the infinite sum term was included in Equation (1), the value of the extinction cross-section with respect to the wavelength did not fluctuate greatly when *n* = 10.

**Figure 6:**
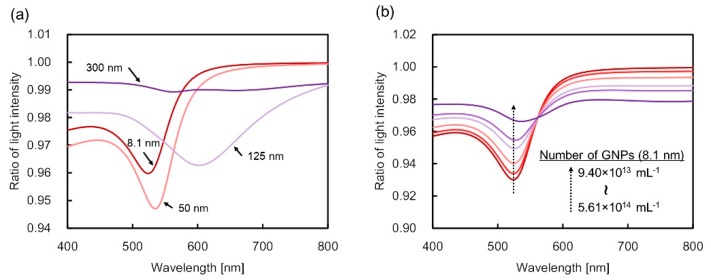
(**a**) Results of numerical calculations of optical extinction spectra accompanying the addition of L-cysteine at 2 wt% of the gold mass concentration. Values in the graph indicate particle diameters of GNPs. The dip value of the extinction spectrum shifts as the GNPs’ diameters in the dispersion solution increase homogeneously. Further, as the particle size increases to more than 125 nm, the number of particles interacting with the optical fiber propagation light decreases, so that the dip of the spectrum tends to decrease. (**b**) Results of numerical calculation by addition of L-cysteine at 4 wt% of mass concentration. The number of nanoparticles in the picoliter cuvette inside the optical fiber is predicted. The particle number of 8.1 nm GNPs decreases while that of the 200 nm GNPs increases. A 520 nm dip wavelength with a particle size of 8.1 nm is decreased. An increase in the 565 nm dip wavelength with a 200 nm particle size cannot be confirmed.

The extinction cross-section approach made it possible to represent the optical extinction spectra by numerical calculation based on Lambert–Beer’s law. The optical path length was assumed to be 3 μm. The particle size was assumed to be 8.1 to 300 nm. The number of GNPs per milliliter was calculated by use of the assumed particle size. The calculated extinction spectra by GNPs were obtained using the optical path length, the number of GNPs per milliliter, and optical extinction cross-section which was calculated from particle parameters. Figure 6a shows the result at a gold mass concentration of 2 wt%. The spectra were calculated under the presumption that the particle size of the nanoparticles in the dispersion solution homogeneously increased after the addition of L-cysteine. Following an increase of the particles’ size, the actual number of particles per unit milliliter in the solution decreased. A shift in the dip wavelength was confirmed to be accompanied by a change in the particle diameter. Notably, as the grain diameter reached approximately 125 nm, the dip value became smaller compared with the case of 8.1 nm. At 0 mM, as in Figure 3a, the number of particles per unit milliliter was assumed to be 3.3 × 10^14^ particles/mL. Nonetheless, the number of particles accompanying the increase in particle diameter decreased to 5.5 × 10^9^ particles/mL as the latter reached 300 nm. The particle size was observed to homogeneously increase in the aggregation process, followed by a decrease in the number of substantial particles interacting with the incident light once it exceeded a certain level. Such behavior resulted in the decreasing tendency of the dip value of the extinction spectrum.

Figure 6b shows the result of optical extinction spectra calculation at a gold mass concentration of 4 wt%, where the number of particles with 8.1 nm particle diameter decreased with the addition of L-cysteine. As the number of 8.1 nm particles was decreased due to aggregation, the reduction of the extinction dip in the vicinity of the 520 nm wavelength was led consequently. At an extinction dip value of 0 mM, as in Figure 3b, the number of particles calculated was 5.61 × 10^14^ particles/mL. Assuming the volume of the picolitter cuvette to be 0.4 pL, the number of GNPs interacting with the light inside the optical fiber was estimated to be 2.24 × 10^5^ particles. As for the other concentrations, the calculated spectra were similar to the experimental results because the number of particles per unit milliliter was estimated from the extinction dip value of the experimental results. In the numerical calculation, the diameter of the GNP after aggregation was set to a representative value of 200 nm. Once L-cysteine reached a concentration of 5 mM, the number of GNPs with a particle size of 8.1 nm decreased to 9.40 × 10^13^ particles/mL, and the number of 200 nm particles increased to 3.10 × 10^10^ particles/mL. In the case of 5 mM of the calculation result, it was confirmed that the dip wavelength was red-shifted due to the increase of GNPs with a particle size of 200 nm. If 5 mM L-cysteine was detected, the amount of L-cysteine was 2.0 × 10^−15^ mol (2.4 × 10^−13^ g) in the picoliter cuvette with the assumption of 0.4 pL sensing volume. Moreover, the linearity of the sensitivity characteristics was confirmed with respect to the added concentration of L-cysteine, as the 4 wt% high concentration GNP dispersion solution was utilized. Additionally, although the numerical calculation reflected 200 nm GNPs, a 565 nm dip could not be identified as shown in Figure 3b, which could be attributed to the size of the few remaining aggregated GNPs compared with the initial size of the GNPs. In the real experiment, the diameter of GNP after aggregation was not fixed at any specific size. The inhomogeneously aggregated particles did not have a particular size, resulting in very little red-shifted extinction. Thus, as a large extinction dip could not be confirmed, the dip obtained in the experimental result was found as the optical extinction spectrum by GNPs that were almost free from aggregation.

## 4. Conclusions

This paper investigated the feasibility of a picoliter cuvette inside an optical fiber to realize a new biosensing measurement configuration that involves the use of a liquid sample. The picoliter sensing volume was obtained via GNP dispersion solution, which induced light attenuation peculiar to the optical extinction spectrum, for a successful demonstration of measuring L-cysteine as a sensing target. During the measurement, it was possible to track the transition of the extinction spectrum by aggregation of the GNPs due to injection of L-cysteine into the dispersion solution. At mass concentration of the GNP dispersion solution of 4 wt%, the measurement resolution for L-cysteine was 0.125 mM in the scale of 0–5 mM. It was revealed that if aggregation is induced in GNPs in solution, it is obtained as a change in the optical extinction spectrum with picoliter sensing volume. Selective sensitivity can be measured by modifying the surface of the GNPs so as to induce aggregation when a specific sensing target is added. As such, biological sensing with selective sensitivity was confirmed as feasible by devising induced aggregation through the introduction of a sensing target into the GNP dispersion solution. Results of the numerical calculations of the optical extinction spectra based on Mie theory, coupled with SEM observation, were consistent with assuming the aggregated particle diameter of GNPs, and the number of particles per unit milliliter. The optical extinction spectra obtained in the experiment indicate the possibility of recursively determining the state of GNPs in the dispersion solution.
